# Evaluation of an organ-based tube current modulation tool in pediatric CT examinations

**DOI:** 10.1007/s00330-020-06888-5

**Published:** 2020-05-20

**Authors:** Antonios E. Papadakis, John Damilakis

**Affiliations:** 1 Department of Medical Physics, University Hospital of Heraklion, Stavrakia, 71110 Heraklion, Crete Greece; 2 Department of Medical Physics, University of Crete, Medical School, Stavrakia, 71110 Heraklion, Crete Greece

**Keywords:** Tomography, x-ray computed, Child, Radiation dosage, Monte Carlo method, Radiation protection

## Abstract

**Objectives:**

To investigate the effect of an organ-based tube current modulation (OTCM) technique on organ absorbed dose and assess image quality in pediatric CT examinations.

**Methods:**

Four physical anthropomorphic phantoms that represent the average individual as neonate, 1-year-old, 5-year-old, and 10-year-old were used. Standard head and thorax acquisitions were performed with automatic tube current modulation (ATCM) and ATCM+OTCM. Dose calculations were performed by means of Monte Carlo simulations. Radiation dose was measured for superficial and centrally located radiosensitive organs. The angular range of the OTCM exposure window was determined for different tube rotation times (*t*) by means of a solid-state detector. Image noise was measured as the standard deviation of the Hounsfield unit value in regions of interest drawn at selected anatomical sites.

**Results:**

ATCM+OTCM resulted in a reduction of radiation dose to all radiosensitive organs. In head, eye lens dose was reduced by up to 13% in ATCM+OTCM compared with ATCM. In thorax, the corresponding reduction for breast dose was up to 10%. The angular range of the OTCM exposure window decreased with *t*. For *t* = 0.4 s, the angular range was limited to 74° in head and 135° for thorax. Image noise was significantly increased in ATCM+OTCM acquisitions across most examined phantoms (*p* < 0.05).

**Conclusions:**

OTCM reduces radiation dose to exposed radiosensitive organs with the eye lens and breast buds exhibiting the highest dose reduction. The OTCM exposure window is narrowed at short *t*. An increase in noise is inevitable in images located within the OTCM-activated imaged volume.

**Key Points:**

*• In pediatric CT, organ-based tube current modulation reduces radiation dose to all major primarily exposed radiosensitive organs.*

*• Image noise increases within the organ-based tube current modulation enabled imaged volume.*

*• The angular range of the organ-based tube current modulation low exposure window is reduced with tube rotation time.*

## Introduction

The lens of the eye and breast are considered among the most radiosensitive tissues of the human body. Based on new epidemiological evidence on the detrimental effects of ionizing radiation, the International Commission on Radiological Protection (ICRP) has recently reduced the radiation dose threshold for cortical and posterior subcapsular cataract formation to 500 mGy [1–4]. ICRP has also increased the breast tissue weighting factor for effective dose estimation from 0.05 to 0.12 [2]. These changes suggest that the lens of the eye and breast may be more radiosensitive than previously considered. Increased attention should thus be given to minimize radiation dose to these tissues, especially in children that are considered more radiosensitive than adults and are more likely to undergo multiple CT examinations during their lifetime [5].

Organ-based tube current modulation (OTCM) techniques reduce the x-ray tube current (mA) over the anterior part of the patient’s body circumference aiming to minimize radiation exposure to superficial radiosensitive organs such as eyes, thyroid, and breasts. Different CT vendors have adopted different approaches to implement OTCM. In one approach, implemented by Siemens Healthcare with X-CARE, mA is reduced when the x-ray tube rotates over the anterior quadrant of the body circumference, while it is increased over the lateral and posterior quadrants to preserve image quality [6–9]. However, an increase in dose absorbed by posterior located radiosensitive organs has been demonstrated [6–8]. In a second approach, implemented by GE Medical Systems with ODM, mA is reduced when the x-ray tube rotates over the anterior part of the patient’s body without increase over the remaining lateral and posterior parts. However, this approach has been documented to deliver images of increased noise [10, 11]. Previous studies performed in adult patients have shown that OTCM may substantially reduce radiation dose to superficial radiosensitive organs [6, 8, 12, 13]. To our knowledge, there is scarce published data on the effect of OTCM on radiation dose to superficial radiosensitive organs and image quality in pediatric CT examinations [14, 15].

The purpose of this study was to investigate the effect of an OTCM technique on radiation dose to major radiosensitive organs and assess image quality in pediatric CT examinations.

## Materials and methods

### Anthropomorphic phantoms

Four physical anthropomorphic phantoms (ATOM Phantoms, CIRS) that simulate the average pediatric individual as neonate, 1-year-old, 5-year-old, and 10-year-old were used [16, 17].

### Organ-based tube current modulation technique

**Fig. 1:**
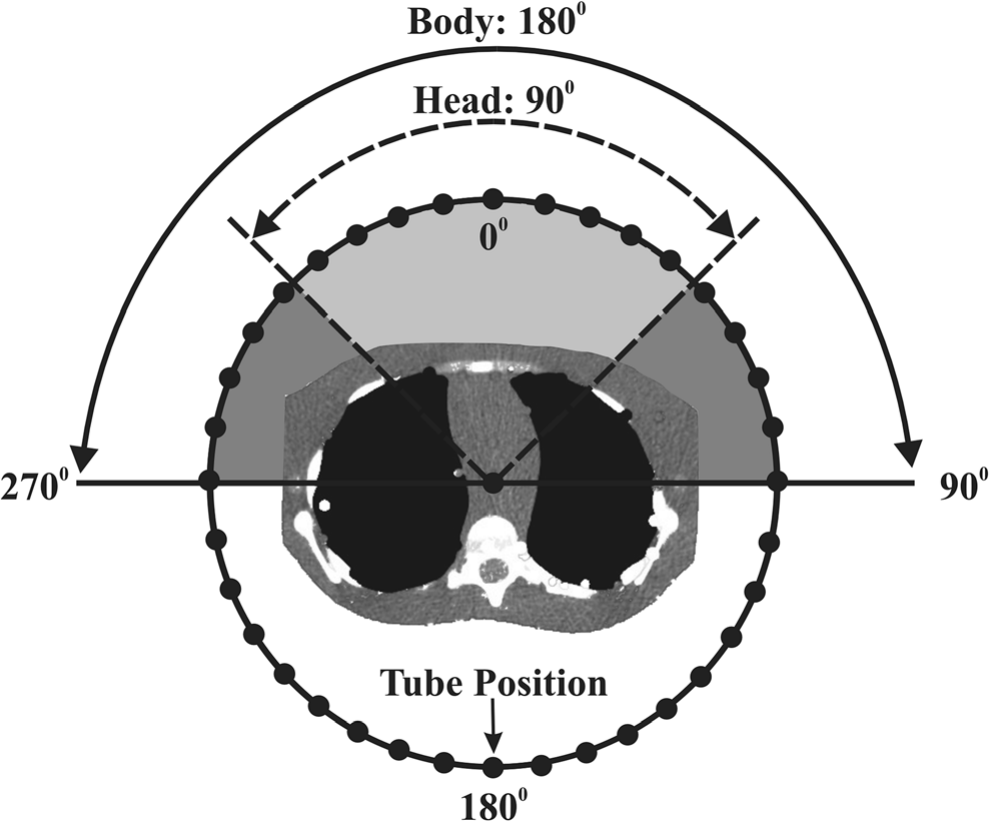
A graphical illustration of the configuration setup used in the MC simulation experiments showing the positioning of the phantom with regard to the angle of OTCM mA reduction. For head, the mA was reduced by 30% in 9 out of 36 tube positions, while for body, the mA was reduced by 40% in 18 out of 36 tube positions

Acquisitions were performed on a 64-detector row CT scanner (Revolution GSI, GE Medical Systems). This scanner is equipped with OTCM (ODM, GE Medical Systems). OTCM constitutes a tube current modulation mode that reduces the mA when the tube travels across the anterior arch of the patient’s circumference without increasing it over the remaining lateral and posterior arches. To enable OTCM, the automatic tube current modulation (ATCM) system (AutomA and SmartmA, GE Medical Systems) needs also to be activated. In head, the mA is reduced by up to 30% across 90° anterior projections, while in body, the mA is reduced by up to 40% across 180° anterior projections (Fig. 1) [18].

### CT examination protocols

Head, thorax, and whole body acquisitions were performed using the scanning parameters listed in Table 1 [19]. Each anatomical region was first scanned with ATCM and then with ATCM+OTCM. All scans were repeated five times to calculate arithmetic averages of the modulated mA values among scans.

**Table 1:** Examination protocols with acquisition and reconstruction parameters for head and thorax pediatric routine CT examinations. All acquisitions were performed in the helical mode. Head acquisitions were performed in the caudocranial direction from the base of the skull to the superior limit of the skull at a pitch of 0.531. Thorax acquisitions were performed in the craniocaudal direction from the apex of the lungs to the end of the diaphragm at a pitch of 1.375. Images were reconstructed using the “standard” reconstruction filter kernel, the adaptive statistical iterative reconstruction (ASIR) at the 40% level, and a slice thickness of 2.5 mm

	Scout view	Scan field of view	kVp	Rot. time (sec)	Beam coll. (mm)	min mA/max mA	Noise index
Head							
Neonate	AP+LAT	Pediatric head	80	0.5	20	100/350	5.80
1 year	AP+LAT	Pediatric head	100	0.5	20	100/350	7.70
5 years	AP+LAT	Small head	120	0.5	20	100/350	5.14
10 years	AP+LAT	Small head	120	1.0	20	100/350	3.86
Thorax							
Neonate	AP+LAT	Pediatric body	80	0.4	20	25/300	11.6
1 year	AP+LAT	Small body	80	0.4	20	25/300	11.6
5 years	AP+LAT	Small body	80	0.4	20	25/300	10.7
10 years	AP+LAT	Large body	80	0.4	40	25/300	13.11

Whole body acquisition was performed using the exposure parameters prescribed for thorax acquisition for each anthropomorphic phantom

### Monte Carlo simulation

**Fig. 2:**
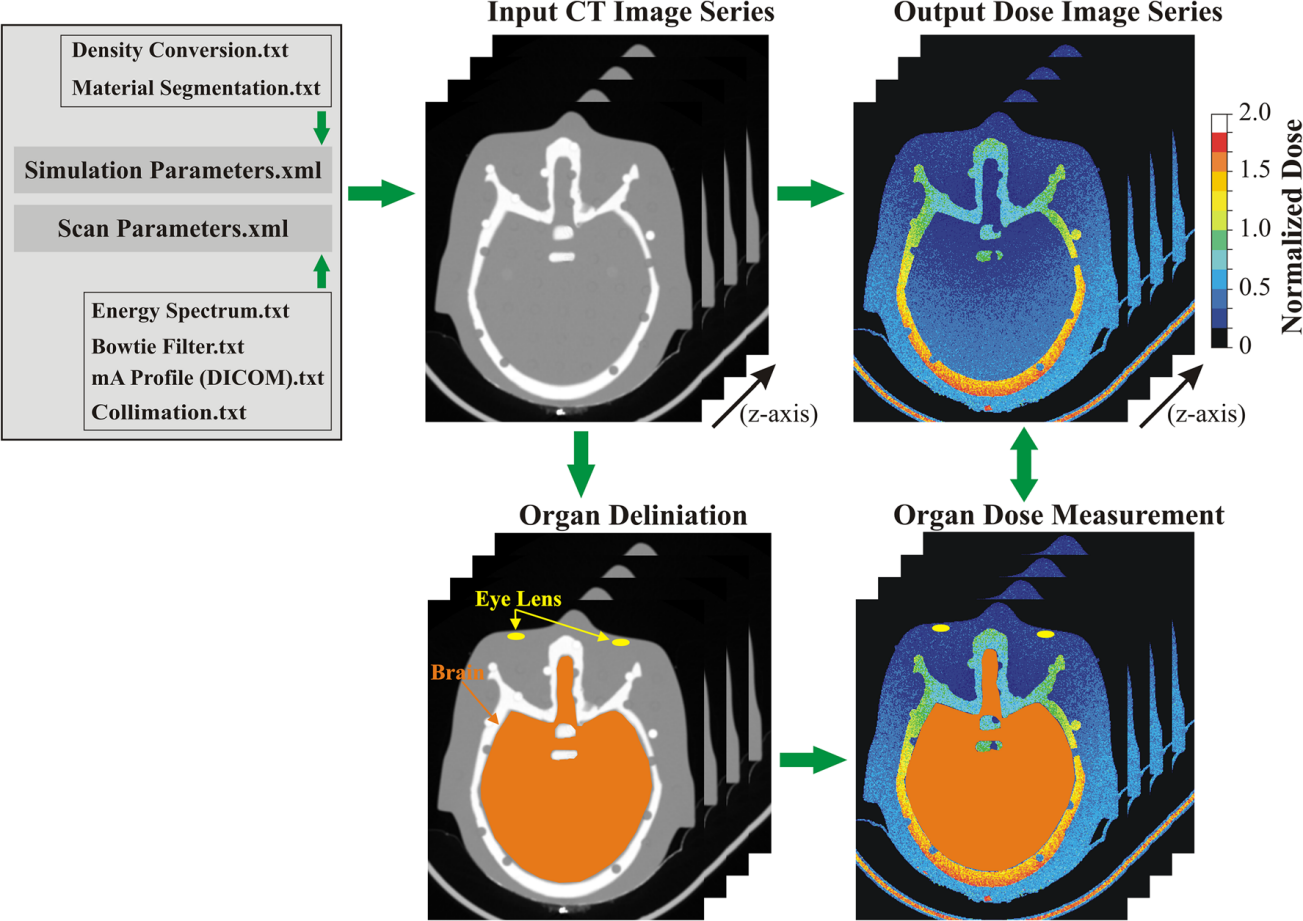
A schematic diagram of the MC simulation algorithm employed. Required data on simulation density conversion and material segmentation along with data on scan parameters including energy spectrum, bowtie filter, mA(z) profile derived from DICOM header, and collimation are input in the form of txt files. Additional parameters such as irradiation geometry, maximum number of simulated photons and energy level below which simulated photons are considered absorbed are also predefined

Three-dimensional radiation dose distributions were generated using a MC simulation tool (ImpactMC, CT Imaging GmbH) [20–23]. Whole body, 2.5 mm thick, CT image series (512 × 512 pixels/image) of the physical phantoms were used as input to create whole body age-specific voxelized phantoms. The employment of whole body voxel phantoms is essential to take into account the contribution of scattered radiation from anatomical regions beyond the imaged volume [20]. Simulations were performed by applying density and material segmentation, and using the scanner geometry, x-ray beam energy spectrum, beam filtration, and geometrical characteristics of small and large bowtie filters for head and thorax, respectively (Fig. 2). Data regarding the geometric characteristics of the bowtie filters were obtained from the manufacturer. Thirty-six tube focus positions were simulated per tube rotation. Acquisitions were simulated from vertex to top of C1 lamina for head, and lung apices to 12th rib for thorax. Simulations were first performed with ATCM and then with ATCM+OTCM. In ATCM, the mA(z) profile derived from the mean mA values listed in the images’ DICOM header was used as input. In ATCM+OTCM, the mA was reduced for the anterior angles that spanned the eyes for head and breast for thorax. For head, mA was reduced by 30% across 9 out of 36 (90°) simulated tube positions. For thorax, mA was reduced by 40% across 18 out of 36 (180°) simulated tube positions. The mA values across the remaining posterior projections were those prescribed by the DICOM header. Following MC simulation, an output color-coded dose image matrix series (512 × 512 pixels/image) was generated. These matrices depict the normalized to free-in-air CTDI_air_ (mGy/mGy·100 mAs) dose distribution (ND_air_) imparted in the phantom’s body, in voxel-to-voxel correspondence to input CT images. Measured dose values were normalized (ND_m_) to CTDI_vol_ (mGy/100 mAs) for 16 cm diameter phantom; ND_m_ = (ND_air_/CTDI_vol_).

To assess the potential effect of altering the angular range of OTCM scheme on organ dose, additional ATCM+OTCM simulations were performed. For head, simulations were performed with the mA being reduced by 30% across 80° and 70°. For thorax, simulations were performed with the mA being reduced by 40% across 160°, 140°, and 120°.

### Organ dose estimation

Organ dose was estimated for eye lens, brain, and salivary glands for head, and breast buds, lung, liver, thyroid and kidneys for thorax. For eye lens, a 30 mm^2^ region of interest (ROI) was drawn in the anterior segment of the left and right eye socket. For breast buds, a 60 mm^2^ ROI was drawn in the anterior chest wall of left and right breast. This ROI size was kept constant for neonate, 1-year-old and 5-year-old phantoms, since breast bud size is considered to be the same until the onset of puberty. Moreover, this size is considered to be identical in boys and girls. To take into account breast development at the onset of girl’s puberty, ROI size was increased to 80 mm^2^ for 10-year-old phantom. ROIs for eye lens and breast buds were drawn in single images considering that these organs are not expected to extend more than 2.5 mm along z-axis. The size and position of all other organs was determined in accordance with previously published data [24]. In head, all examined organs were fully encompassed in the simulated scanned volume. In thorax, breast buds and lungs were fully encompassed; thyroid and kidneys were outside, while liver was partially encompassed in the simulated scanned volume. ROIs per input CT image slice (ROI(i)) were transferred to the corresponding output dose matrices. The mean dose image pixel (MDP(i)) and its standard deviation (SD) from each ROI were recorded. MDP(i) was the dose absorbed for the fraction of the organ depicted in that particular image. The total organ dose (TOD) was estimated as the sum of the area-weighted average of MDP(i) values obtained from all images where the organs were depicted;

1 $$ \mathrm{TOD}=\left(\frac{\sum \mathrm{MDP}(i)\times \mathrm{ROI}(i)}{\sum \mathrm{ROI}(i)}\right) $$

To take into account the error in organ dose measurement related to the location of each radiosensitive organ, each ROI was drawn ten times at slightly different locations along the x-y plane of the axial image. The error in estimated organ dose was calculated through error propagation using the standard deviation of the average measured organ doses and the recorded SDs of the MIP(i) values. For comparison of estimated doses, the Student’s *t* test for paired samples was used. A significant difference was set at *p* < 0.05.

### OTCM: the effect of tube rotation time (*t*) on tube output

To investigate the potential effect of *t* on OTCM scheme, the tube output was measured using an x-ray multimeter equipped with a solid-state point detector (Black Piranha, CT Dose Profiler, RTI Electronics). This detector is capable of acquiring up to 2000 exposure measurements/s. ATCM+OTCM acquisitions were performed at 0.4, 0.5, 0.7, 1, and 2 s. The standard head, 16 cm diameter, polymethyl methacrylate (PMMA) phantom (CT-Phantom, IBA Dosimetry) was scanned using the examination protocols applied for pediatric head and thorax under fixed mA, ATCM, and ATCM+OTCM (Table 2). This phantom was selected because it is cylindrical, provides uniform attenuation across 360° and is used as reference for CTDI_vol_ reporting in pediatric patients. In fixed mA acquisition, the mA was manually adjusted to reach a CTDI_vol_ that matches the corresponding CTDI_vol_ of ATCM-activated acquisition. AP and LAT scout views of the phantom were acquired. The phantom was then removed from the table and the solid-state detector was positioned, free-in-air, at the gantry isocenter. A floor-mounted arm was used to keep the detector stationary throughout the scan. Exposure rate profiles as a function of tube projection angle were recorded. This configuration facilitates free-in-air measurement of the tube output based on the scout view predetermined mA-modulated profiles.

**Table 2:** Examination protocol parameters used to measure tube output free-in-air under three operation modes with fixed mA, ATCM, and ATCM+OTCM exposure of the standard, 16 cm diameter, head PMMA phantom

	Scout view	Scan field of view	Beam coll. (mm)	Rot. time (sec)	kVp	Operation mode	
Head							
	AP+LAT	Pediatric head	20	0.4	120	Fixed mA	180 mA
						ATCM	NI: 4.5
						ATCM+OTCM	NI: 4.5
Thorax							
	AP+LAT	Pediatric body	20	0.4	80	Fixed mA	35 mA
						ATCM	NI: 14
						ATCM+OTCM	NI: 14

### Quantitative image quality assessment

**Fig. 3:**
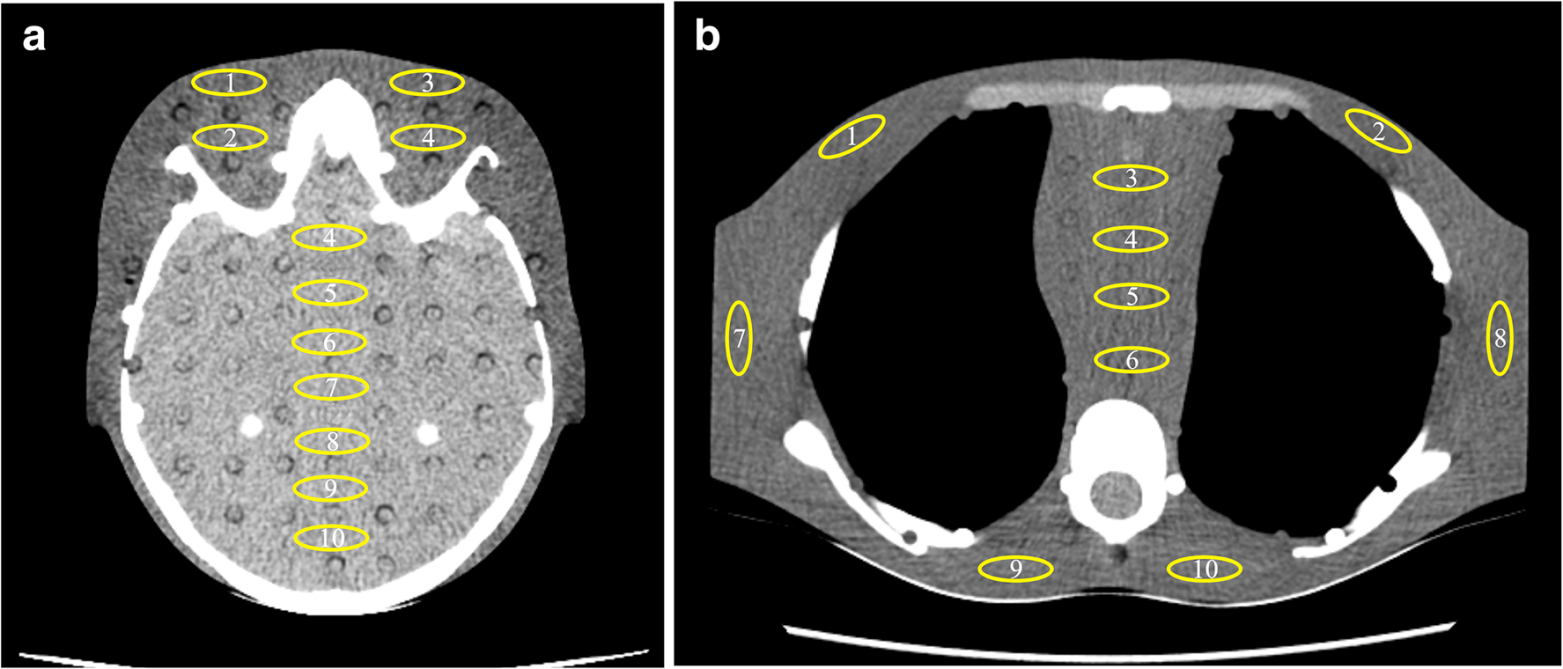
ROI placement for image noise measurement in ATCM and ATCM+OTCM-activated head (**a**) and thorax (**b**) acquisitions. Head and thorax images shown are from the 10-year-old phantom. Window width 100 and window level 30 for (**a**); window width 400 and window level for 40 (**b**)

To assess the effect of OTCM on image quality, we have compared the noise in images obtained from ATCM and ATCM+OTCM-activated acquisitions. Image noise was measured as the SD of the mean Hounsfield unit (HU) value in ~ 3 cm^2^ ROIs. These ROIs were drawn at various locations over uniform brain and soft tissue equivalent areas (Fig. 3). Ten ROIs were drawn in the images obtained with ATCM at the level depicting eyes for head and middle heart for thorax. These ROIs were pasted to the corresponding images obtained with ATCM+OTCM. To reduce measurement error, each parameter was measured five times on five consecutive images. Quantitative image analysis was performed using ImageJ (1.48v, NIH). For noise comparison, the Student’s *t* test was used for paired samples. A significant difference was set at *p* < 0.05.

## Results

In ATCM-activated head acquisitions, the absorbed dose to the eye lens ranged from 3.6 mGy for neonate to 27.6 mGy for 10-year-old phantom (Table 3). The corresponding absorbed doses in ATCM+OTCM-enabled acquisitions ranged from 3.1 to 25.0 mGy, resulting in 13% and 9% reduction, respectively. In ATCM-activated thorax acquisitions, the absorbed dose to the breast buds ranged from 0.55 mGy for neonate to 1.3 mGy for 5-year-old phantom. The corresponding absorbed doses in ATCM+OTCM-enabled acquisitions ranged from 0.53 to 0.95 mGy, resulting in 4% and 27% reduction, respectively. Organ dose was slightly increased when the angular range of the OTCM scheme was reduced, with this increase, however, being not statistically significant (Table 4).

**Table 3:** CTDI_vol_ and calculated organ dose values (mGy) in ATCM and ATCM+OTCM-activated head and thorax CT acquisitions. Numbers in parenthesis are percent organ dose reduction

CTDI_vol_ (mGy) and organ dose (mGy), ATCM*/ATCM+OTCM				
	Neonate	1-year-old	5-year-old	10-year-old
Head				
CTDI_vol_	11.34/10.86	16.67/16.25	24.62/24.04	49.53/47.83
%Diff	− 4%	− 2%	− 2%	− 3%
Eye lens	3.6 ± 0.06/3.1 ± 0.05	9.0 ± 0.16/7.7 ± 0.14	18.7 ± 0.3/17.0 ± 0.3	27.6 ± 0.5/25.0 ± 0.4
%Diff/*p* value	− 14%/< 0.001	− 14%/< 0.001	− 9%/< 0.001	− 9%/< 0.001
Brain	3.5 ± 0.17/3.3 ± 0.05	9.3 ± 0.46/9.0 ± 0.45	22.6 ± 1.1/22.0 ± 1.1	41.2 ± 2.0/38.9 ± 1.9
%Diff/*p* value	− 6%/< 0.05	− 3%/0.157	− 3%/0.238	− 5%/< 0.05
Salivary glands	3.7 ± 0.07/3.4 ± 0.07	10.5 ± 0.21/9.4 ± 0.19	24.6 ± 0.5/22.2 ± 0.4	36.3 ± 0.7/33.6 ± 0.7
%Diff/*p* value	− 8%/< 0.001	− 10%/< 0.001	− 10%/< 0.001	− 7%/< 0.001
Thorax				
CTDI_vol_	1.87/1.70	1.46/1.33	2.06/1.94	2.82/2.73
%Diff	9%	9%	6%	3%
Breast buds	0.55 ± 0.009/0.53 ± 0.009	1.1 ± 0.02/0.79 ± 0.01	1.3 ± 0.02/0.95 ± 0.016	1.1 ± 0.02/1.0 ± 0.02
%Diff/*p* value	− 4%/0.097	− 28%/< 0.001	− 27%/< 0.001	− 9%/< 0.001
Lung	0.53 ± 0.039/0.47 ± 0.035	0.95 ± 0.070/0.85 ± 0.063	1.5 ± 0.11/1.4 ± 0.10	1.3 ± 0.09/1.2 ± 0.09
%Diff/*p* value	− 11%/< 0.05	− 10%/< 0.05	− 7%/< 0.05	− 8%/< 0.05
Liver	0.43 ± 0.048/0.39 ± 0.043	0.85 ± 0.094/0.79 ± 0.088	0.63 ± 0.069/0.61 ± 0.065	1.1 ± 0.12/1.0 ± 0.11
%Diff/*p* value	− 9%/0.065	− 7%/0.157	− 3%/0.513	− 9%/0.067
Thyroid	0.25 ± 0.014/0.22 ± 0.01	0.46 ± 0.026/0.44 ± 0.025	0.27 ± 0.015/0.25 ± 0.014	0.28 ± 0.016/0.26 ± 0.015
%Diff/*p* value	− 12%/< 0.001	− 4%/0.096	− 7%/< 0.05	− 7%/< 0.05
Kidneys	0.25 ± 0.028/0.23 ± 0.026	0.15 ± 0.017/0.14 ± 0.016	0.16 ± 0.018/0.16 ± 0.017	0.68 ± 0.076/0.61 ± 0.069
%Diff/*p* value	− 8%/0.115	− 6%/0.192	–	− 10%/< 0.05

*As the mean mA value listed in the images’ DICOM header per z-axis location is averaged over the transversal plane, the resulting mA(z) profile will represent rather the longitudinal (auto mA) than the transversal (smart mA) mA profile. Moreover, the anterior part of the transversal mA profile will be overestimated through the mean mA(z) profile, and the lateral part of the transversal mA profile will be underestimated through the mean mA(z) profile. Due to the anthropometry of children, this effect increases with the child’s age. However, the relative organ dose values listed herein will remain largely unaffected

**Table 4:** Calculated organ dose values (mGy) in ATCM+OTCM-activated head and thorax CT acquisitions of the 5-year-old anthropomorphic phantom at different OTCM schemes. Similar results were found for all anthropomorphic phantoms

Organ dose (mGy), ATCM+OTCM				
	90°	80°	70°	–
Head				
Eye lens	17.0 ± 0.3	17.0 ± 0.3	17.2 ± 0.3	**–**
Brain	22.0 ± 1.1	22.2 ± 1.0	22.2 ± 1.1	**–**
Salivary glands	22.2 ± 0.4	22.2 ± 0.4	22.3 ± 0.3	**–**
	180°	160°	140°	120°
Thorax				
Breast buds	0.95 ± 0.016	0.96 ± 0.014	0.96 ± 0.012	0.96 ± 0.016
Lung	1.4 ± 0.10	1.4 ± 0.11	1.5 ± 0.12	1.6 ± 0.11
Liver	0.61 ± 0.065	0.61 ± 0.075	0.63 ± 0.071	0.64 ± 0.070
Thyroid	0.25 ± 0.014	0.26 ± 0.014	0.25 ± 0.014	0.26 ± 0.014
Kidneys	0.16 ± 0.017	0.16 ± 0.019	0.17 ± 0.016	0.16 ± 0.020

**Fig. 4:**
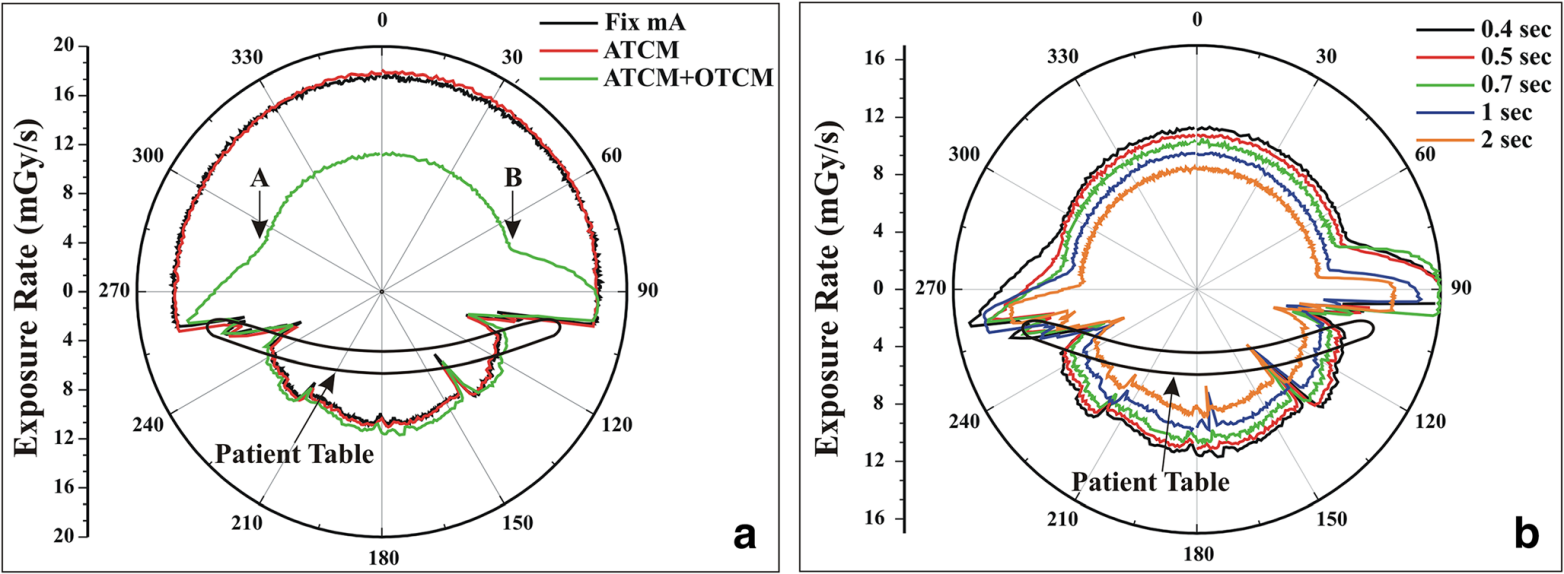
**a** Measured exposure rate as a function of tube projection angle in fixed mA, ATCM, and ATCM+OTCM acquisition of the 16 cm diameter, PMMA phantom using the thorax examination protocol. Tube rotation time was set at 0.4 s. The low exposure rate is applied across a 140° arch (from point (A) to (B)), while on either side of the low exposure window, there is a 30° transition range. Exposure rate does not differ between fixed mA and ATCM due to the circular cross section of the PMMA phantom and the exposure geometry. The abrupt exposure rate changes across the 90° to 270° arch originate from changes in attenuation of the patient’s table top across tube projections. **b** Measured exposure rate as a function of tube projection angle in ATCM+OTCM acquisition of the 16 cm diameter, PMMA phantom at different (*t*) for thorax examination protocol

Measurements of exposure rate on the 16 cm diameter PMMA phantom confirmed that OTCM reduces exposure (at 0°) by 30% in head and 40% in thorax. In head, the angular range of OTCM window increased from 74° for 0.4 s to 95° for 2 s. In thorax, the corresponding increase was from 135° to 170°. Shown in Fig. 4 for thorax is the measured exposure rate as a function of projection angle in fixed mA, ATCM, and ATCM+OTCM acquisitions (a) and the ATCM+OTCM-activated exposure rate profiles at different *t* (b).

**Fig. 5:**
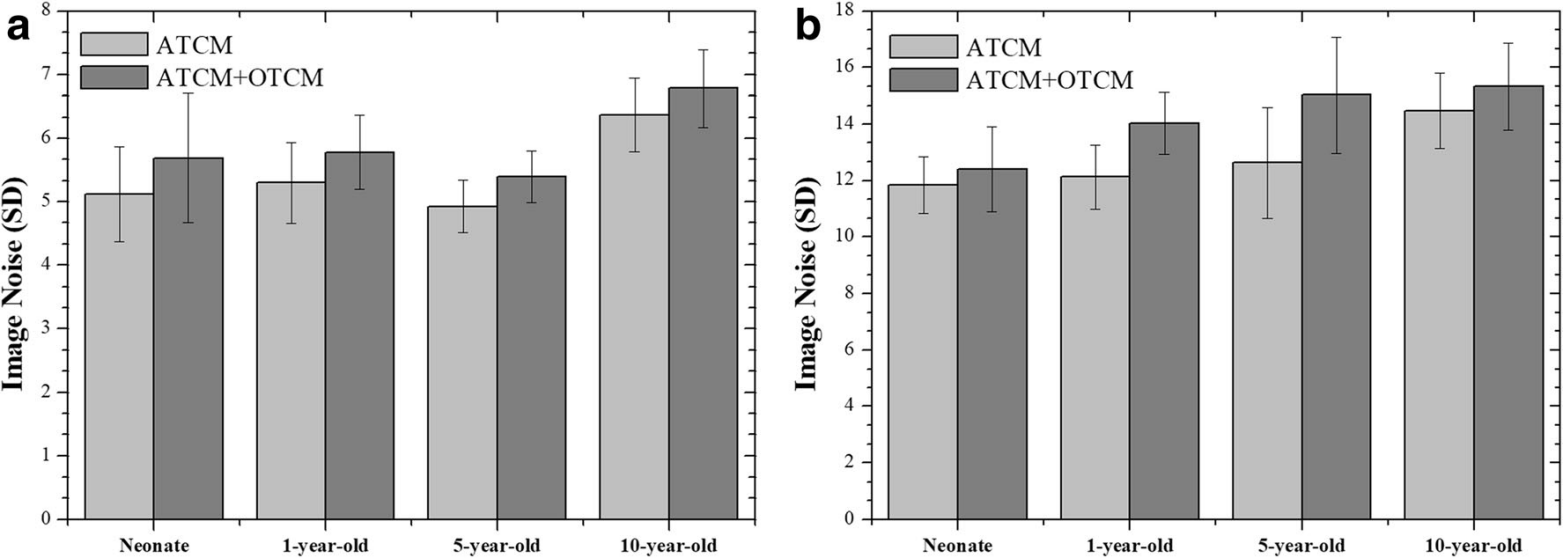
Mean image noise values averaged over ROI 1 through ROI 10 at each anthropomorphic phantom for head (**a**) and thorax (**b**) acquisitions

Image noise was increased in ATCM+OTCM-enabled acquisitions (Table 5). Mean image noise increase was up to 11% for head and 19% for thorax (Fig. 5). The increase in image noise was statistically significant (*p* < 0.05) in all but neonate phantom.

**Table 5:** Image noise values measured in ROIs 1 through 10 in ATCM and ATCM+OTCM acquisitions for head and thorax. The location of ROIs is demonstrated in Fig. 1

	ATCM/ATCM+OTCM (%Diff)			
ROI	Neonate	1-year-old	5-year-old	10-year-old
Head				
1	4.21/4.47 (6%)	5.01/5.36 (7%)	5.04/4.85 (− 3%)	5.58/5.66 (1%)
2	4.86/4.35 (− 10%)	5.51/6.09 (10%)	5.23/5.83 (11%)	5.86/5.91 (1%)
3	4.96/4.64 (− 6%)	6.24/6.86 (10%)	4.83/5.39 (11%)	7.42/7.68 (3%)
4	4.21/5.51 (30%)	6.38/6.02 (− 5%)	5.66/6.03 (6%)	6.40/6.72 (5%)
5	6.07/7.34 (21%)	5.69/6.35 (11%)	5.24/5.58 (6%)	6.68/6.96 (4%)
6	5.60/6.78 (21%)	5.07/5.88 (16%)	4.98/5.49 (10%)	6.37/6.99 (9%)
7	6.12/5.74 (− 6%)	4.74/5.42 (14%)	4.88/5.22 (7%)	5.97/6.58 (10%)
8	5.89/6.19 (5%)	4.95/5.17 (4%)	4.47/5.00 (11%)	6.37/7.22 (13%)
9	4.76/5.39 (13%)	4.87/5.71 (17%)	4.70/4.85 (3%)	5.87/6.84 (16%)
10	4.48/6.45 (43%)	4.49/4.91 (9%)	4.20/5.64 (34%)	7.117.25 (2%)
Thorax				
1	9.89/9.79 (− 1%)	10.79/12.88 (19%)	10.99/12.50 (13%)	12.44/12.59 (1%)
2	10.69/10.05 (− 6%)	12.54/12.79 (2%)	8.66/12.39 (43%)	12.64/13.01 (3%)
3	12.36/11.98 (− 3%)	10.01/12.36 (23%)	13.29/15.34 (15%)	13.72/15.92 (16%)
4	12.33/13.61 (10%)	10.88/14.40 (32%)	12.90/17.59 (36%)	14.56/14.62 (0%)
5	12.43/11.97 (− 3%)	12.72/14.85 (16%)	15.17/16.71 (10%)	14.36/16.54 (15%)
6	12.36/13.69 (10%)	12.77/14.90 (16%)	14.66/16.72 (14%)	16.37/16.45 (0%)
7	13.33/13.72 (3%)	12.36/13.88 (12%)	12.02/12.82 (6%)	14.00/14.87 (6%)
8	11.49/13.72 (19%)	12.93/14.13 (9%)	11.29/13.13 (16%)	14.72/15.69 (6%)
9	12.14/13.34 (9%)	12.70/14.10 (11%)	12.99/16.46 (26%)	15.80/16.55 (5%)
10	11.26/12.22 (8%)	13.53/15.91 (17%)	14.24/16.49 (15%)	16.01/17.05 (6%)

## Discussion

To our knowledge, this is the first study aimed to assess the effect of OTCM on radiation dose of radiosensitive organs in children. The presented results showed that OTCM reduces radiation dose to all examined radiosensitive organs and that the angular range of the OTCM low exposure window varies on *t*. While nominal angular ranges prescribed by the manufacturer are 90° for head and 180° for thorax, the results of the current study showed that for *t* = 0.4 s, the low exposure window may be limited to 74° for head and 135° for thorax. This suggests that some organs might benefit less from the reduced angular range of the OTCM window. Our results showed however that, although organ dose was slightly increased, this increase was not statistically significant.

Several studies have demonstrated the effect of OTCM on organ dose in CT of adults. In most studies, investigators have employed an OTCM technique that reduces the mA by 20% for the anterior 120° projection views, while increases the mA for the remaining projections, so that the total exposure during one tube rotation is equal to the exposure without OTCM [25]. This increase is required to preserve image quality. Although this approach has been documented to reduce dose to superficial radiosensitive organs, it provokes a dose increase to centrally and posteriorly located organs. Franck et al [6] have shown that OTCM in thorax CT of females reduces the dose to thyroid and breast by 18% and 19%, respectively. However, the doses for lung, liver, and kidney were found 17%, 11%, and 26% higher. Ketelsen et al have shown that OTCM reduces breast dose by 35% without a change in signal-to-noise ratio [26]. Wang et al, in a study using a non-anthropomorphic phantom, have shown that OTCM reduces breast dose by 12% at an increased, however, CTDI_vol_ [27]. Yamauchi-Kawaura et al have reported that OTCM reduces eye lens and breast dose by 26% and 17%, respectively, in a 6-year-old anthropomorphic phantom, but increases posterior skin dose by 20% [15].

Herein, an OTCM technique that reduces the mA for the anterior without increasing it in the remaining posterior views has been employed. That technique was shown to reduce radiation dose not only to superficial but also to more centrally located organs. The effect of OTCM on radiation dose and image quality using this technique has been presented in two studies. These studies, however, have been limited to adults. Gandhi et al have shown that dose is reduced by 20% in eye lens and 8% in brain for head, and 34% in breast, 20% in lung, and 8% in spine for thorax [10]. This reduction was associated with a 6–20% increase in image noise. Dixon et al, in a study using non-anthropomorphic phantoms, have shown that CTDI_vol_ is reduced with an associated increase in image noise [11]. Herein, the dose to superficial and centrally located radiosensitive organs has been estimated using pediatric anthropomorphic phantoms. OTCM provokes a reduction in dose of all examined organs. This reduction is more significant for organs located within the OTCM-enabled image volume (Table 3). Large organs, such as lungs, in larger compared with smaller body sizes may fall partially outside the OTCM window. It would thus be reasonable to assume that dose for such organs might be less reduced. However, a trend of organ dose reduction with body size was not observed herein. This may be partly attributed to the different exposure parameters used for each anthropomorphic phantom and the limited number of examined body sizes.

It should be noted that image quality should be thoroughly evaluated when dose reduction strategies are employed. Our results on noise measurements in slices within the OTCM-enabled image volume showed that OTCM provokes a statistically significant but quantitatively small increase in noise (Table 5). Of note is that the %Difference in noise varies strongly across the axial slice. There are also few ROIs where noise is reduced. Image noise increase might be higher in ROIs located in anterior locations of the slice where mA is reduced. However, such a trend was not observed herein. This is owing to the statistical behavior of noise, which may prevail over its dependence on mA. Moreover, although the examined ROIs contain uniform soft tissue equivalent material, they are in close proximity with high attenuating bone structures which may affect the recorded noise through beam hardening. Gandhi et al, in an phantom study, have simulated a virtual ATCM+OTCM mode that employs the mA modulation scheme for the anterior 90° or 180° views, as prescribed by the OTCM used herein, but increases the mA for the remaining views [10]. As expected, this mode reduced dose for eye lens and breast to a lower, however, extent relative to real OTCM. It provided similar dose for lung and brain, and increased dose for spine. However, the increase in posterior mA did not fully recover image noise [10]. Whether or not the quantitatively small noise increase in images located within the OTCM-enabled volume is acceptable in the clinical practice is a matter of a further patient study. Of note is that image noise throughout the remaining image volume is expected to remain unaltered given that the applied mA values throughout this volume are governed by the ATCM modulation scheme, which is identical between the ATCM and ATCM+OTCM. Patient dose might also be reduced in the ATCM mode by simply decreasing CTDI_vol_. For instance, to achieve the image noise increase of 15% found for thorax of the 1-year-old phantom, CTDI_vol_ might be reduced by [1-(1/1.15)^2^]×100% = 24%, which is considerably higher compared with 9% achieved with the ATCM+OTCM mode (Table 3). However, it should be emphasized that the 15% noise increase refers to every image within the series of the scanned volume.

This study has some limitations. First, clinical image quality was not evaluated. A further clinical study on a large number of pediatric patients at various ages and body sizes is required to investigate the effect of ATCM+OTCM on image quality. Second, image quality was assessed only on the image noise measure. A subjective evaluation of image quality from experienced pediatric radiologists is needed to assess the effectiveness of OTCM on generating images of diagnostic quality. Third, this study was limited to a single OTCM technique, which is available in the CT unit installed in our hospital.

In conclusion, our results showed that OTCM reduces radiation dose to all examined radiosensitive organs over the range of pediatric anthropomorphic phantoms. Eye lens in head and breast buds in thorax exhibit the highest dose reduction. However, OTCM increases image noise within the OTCM-enabled imaged volume. Besides, we have characterized the tube radiation exposure when the OTCM acquisition mode is enabled in head and thorax of pediatric CT. Our results have shown that the low exposure window is substantially narrowed when short *t* is used.

## Abbreviations

ASIR: Adaptive statistical iterative reconstruction
ATCM: Automatic tube current modulation
CTDI_vol_: Volume computed tomography dose index
DICOM: Digital Imaging and Communications in Medicine
MC: Monte Carlo
OTCM: Organ-based tube current modulation
PMMA: Polymethyl methacrylate
ROI: Region of interest
*t*: Tube rotation time
